# Longitudinal change in physical activity and adiposity in the transition from adolescence to early adulthood: the 1993 Pelotas cohort study

**DOI:** 10.1186/s12966-022-01321-0

**Published:** 2022-07-14

**Authors:** Soyang Kwon, Ana M. B. Menezes, Ulf Ekelund, Fernando C. Wehrmeister, Helen Gonçalves, Bruna Gonçalves C. da Silva, Kathleen F. Janz

**Affiliations:** 1 Ann & Robert H. Lurie Children’s Hospital of Chicago, 225 E Chicago Ave. Box 157, Chicago, IL 60611 USA; 2 Post-Graduate Program in Epidemiology, Federal University of Pelotas, R Marechal Deodoro, 1160 – 3rd floor,, Pelotas, RS CEP 96020-220 Brazil; 3 Department of Sports Medicine, Norwegian School of Sports Sciences, Oslo, Norway; 4 Department of Chronic Diseases, Norwegian Institute of Public Health, Postboks 4014 Ulleål Stadion, 0806 Oslo, Norway; 5 Norwegian Institute of Public Health, Oslo, Norway; 6 Department of Health and Human Physiology, University of Iowa, 102 E FH, Iowa City, 52242 USA

**Keywords:** Physical activity guidelines, Youth, Accelerometers, DXA, Obesity, Fat mass

## Abstract

**Background:**

In the current Physical Activity Guidelines (PAG) for moderate- and vigorous-intensity physical activity (MVPA), abrupt transition from ≥ 60 min/day [youth PAG] to ≥ 150 min/week (≥ 22 min/day on average) [adult PAG] during emerging adulthood is poorly justified. The aim of this study was to examine body fat mass changes according to whether meeting the youth and adult PAGs in late adolescence (age 18 years) to early adulthood (age 22 years).

**Methods:**

The study sample included 2,099 participants (1,113 females) from the 1993 Pelotas (Brazil) Study. At ages 18 and 22 years, MVPA was measured using wrist-worn accelerometry and fat mass was measured using dual-energy X-ray absorptiometry. MVPA at age 18 was categorized into two groups: 0–59 or ≥ 60 min/day (no [N] or yes [Y] for meeting the youth recommendation, respectively). MVPA at age 22 was categorized into three groups: 0–21, 22–59, or ≥ 60 min/day (N, Y22, or Y60 for not meeting the adult recommendation, meeting the adult recommendation, or meeting the youth recommendation, respectively). The combination of these groups created six MVPA groups (N&N, N&Y22, N&Y60, Y&N, Y&Y22, and Y&Y60). Sex-specific multivariable linear regression analyses were conducted to estimate change in fat mass index (FMI) from age 18 to 22 years in the six MVPA groups.

**Results:**

Among males, compared to Y&Y60 (FMI increase = 1.2 kg/m^2^ [95% CI = 1.0, 1.4]), Y&Y22 and Y&N had larger FMI increases (1.9 [1.6, 2.1] and 1.9 [1.2, 2.5], respectively). Among females, Y&Y60 and Y&Y22 had an equal FMI increase (1.6 [1.4, 1.9] for both groups), while Y&N had a larger FMI increase (2.4 [1.8, 3.0]).

**Conclusions:**

These findings suggest that among those who were active in late adolescence, engaging in ≥ 22 min/day of MVPA in adulthood is associated with lower body fat gain for females, but not for males.

**Supplementary Information:**

The online version contains supplementary material available at 10.1186/s12966-022-01321-0.

## Background

Physical activity (PA) provides numerous health benefits across the life course [1, 2]. However, physical inactivity is prevalent globally [3, 4]. To promote and support healthy levels of PA across various populations, the World Health Organization (WHO) [1] as well as several developed countries, such as Australia [5], Canada [6], United Kingdom [7], and United States [2], have established physical activity guidelines (PAG). The guidelines are organized using a lifecourse approach with specific recommendations for youth, adults, and older adults. The recommendations for moderate- and vigorous-intensity PA (MVPA) are overall consistent across the guidelines, which recommend ≥ 60 min/day for youth age 5 to 17 or 18 years (youth PAG) and ≥ 150 min/week (≥ 22 min/day on average) for adults (adult PAG). Notably, the transition in recommended MVPA level abruptly changes from late adolescence to emerging adulthood (18–25 years of age) [8], dropping from ≥ 60 min/day to ≥ 22 min/day [9, 10].

The emerging adulthood population is considered to be one of the healthiest age subgroups, and as such, they have often been overlooked in studied of physical health and health behaviors [11]. However, recent statistics are alarming: one in four U.S. young adults age 19 to 34 years is estimated to have prediabetes, with males having a two-fold higher prevalence than females [12]. Obesity plays a major role in the development of prediabetes and the later development of type 2 diabetes and cardiovascular diseases [12]. During emerging adulthood, obesity incidence is higher compared to the adolescent period [13–15] or any other adulthood periods [16].

A knowledge gap exists regarding the dose–response relationship between MVPA and adiposity during this transitional period. Oh et al. [17] examined the relationship between MVPA patterns from age 15 to 23 years and fat mass at age 23 years and found that participants with consistently higher MVPA (approximately 100 min/day) from age 15 to 23 years had lower fat mass at age 23 years compared to those with lower and declining MVPA (approximately 65 min/day at age 15 years to approximately 50 min/day at age 23 years). The study [17] provided valuable insight that maintaining higher MVPA during the transitional period is associated with lower body fat in early adulthood. However, the study was limited by a relatively small sample size and did not examine adiposity development separately for those who meet the youth PAG and/or the adult PAG and for those who do not.

Few studies have prospectively examined the associations between PA and body fat during the transition from adolescence to young adulthood, a period when the current PAG changes abruptly [9]. Although obesity is a health risk shared by low-, middle-, and high-income countries [18], studies in low- and middle-income countries (LMIC) are particularly limited [19]. The data that are available suggest that the influences of societal factors on PA and adiposity are different in LMIC compared to high income countries [20]. The aim of this study was to compare changes in body fat mass according to whether meeting the youth and adult PAGs from late adolescence to early adulthood (age 18 to 22 years) in a large sample of the Brazilian population. There was no prespecified hypothesis.

## Methods

### Study sample

A study design was a prospective cohort study design. The study sample were participants of the 1993 Pelotas (Brazil) Study [21]. The 1993 Pelotas study was a birth cohort study that recruited 5,249 newborns (99.7% of all births) in the city of Pelotas, Brazil, in 1993 and followed them over time. For an assessment at age 18 years (2011 and 2012), 4,563 cohort members were located, of whom 4,106 (90.0%) attended the clinic visit. For an assessment at age 22 years (2015 and 2016), 4,933 cohort members were located, of whom 3,810 (77.2%) attended the clinic visit. Participants were ineligible for accelerometry assessment if participants did not live in Pelotas at the time of data collection, had physical disabilities, or were unable to wear an accelerometer due to work restriction. Participants were ineligible for dual-energy X-ray absorptiometry (DXA) assessment if participants used a wheelchair or had osteoarticular deformities, implanted metal pins, screws, plates and non-removable metallic objects (body piercings and/or chains), or were extreme obesity, height over 1.92 m, or pregnant [22]. The current report used the data from 1993 Pelotas study participants who completed accelerometer assessments and DXA assessments at 18 and 22 years of age. All study protocols were approved by the Ethics Committee of the Federal University of Pelotas Medical School (register number 05/2011 and 1.250.366). Written informed consent was obtained from individual participants.

### Exposure

The exposure of interest was change in accelerometry-measured MVPA from age 18 to 22 years. Because of device availability, different brands of accelerometers were utilized: GENEActiv Accelerometers (range of ± 8 g; 85.7 Hz; 5-s epoch; ActivInsights, Kimbolton, UK) at age 18 and ActiGraph GT3X + (range of ± 8 g; 60 Hz; 5-s epoch; ActiGraph Inc., Pensacola, FL, USA) at age 22. A prior study [23] showed a high agreement for an acceleration magnitude metric (intraclass correlation coefficient for Euclidian Norm Minus One [ENMO] = 0.99) between the two devices. During clinic visits at age 18 and 22 years, participants were asked to wear an accelerometer on the non-dominant wrist for 24 h and for 4 to 7 consecutive days, including at least 1 weekend day. Detailed information regarding the protocol can be found elsewhere [19, 24].

Accelerometer data were analyzed using the R-package GGIR [25]. Briefly, the GGIR data processing includes automatic calibration, detection of sustained abnormally high values, detection of non-wear, detection of wake/sleep, and quantification of dynamic acceleration magnitude. Data were analyzed per 24 h from midnight to midnight (MM windows). To be included in data analysis, at least 3 wear days and at least 16 wear hours per day were required [26]. The activity-related acceleration metric, ENMO, was calculated. ENMO (m*g*) is one omnidirectional measure of body acceleration, calculated by subtracting the value of gravity from vector magnitude [√(x^2^ + y^2^ + z^2^)-1] [27]. MPA minute was defined as a minute with 100 mg ≤ ENMO ≤ 400 mg for ≥ 48 s (80% of 60 s) [19]. VPA minute was defined as a minutes with ENMO ≥ 400 mg for ≥ 48 s [19]. MVPA minutes were calculated by summing MPA minutes and VPA minutes. In addition, because in the adult PAG (i.e., ≥ 150 min/week of MPA, ≥ 75 min/week of VPA, or an equivalent combination of MPA and VPA) [1, 2], one minute of VPA is considered to be equivalent to two minutes of MPA, we calculated MPA-equivalent minutes (minutes/day) at age 22 years by summing MPA minutes and twice VPA minutes (MPA minutes + 2 × VPA minutes) [28].

MVPA at age 18 years was categorized into two groups: 0–59 or ≥ 60 min/day (no [N] or yes [Y] for meeting the youth PAG). MVPA at age 22 years was categorized into three groups: 0–21, 22–59, or ≥ 60 min/day (N, Y22, or Y60 for not meeting the adult PAG, meeting the adult PAG, or meeting the youth PAG, respectively). The combination of these groups created six MVPA groups (N&N, N&Y22, N&Y60, Y&N, Y&Y22, or Y&Y60). In addition, we also created six weighted-MVPA (wMVPA) groups using MPA-equivalent minutes, instead of MVPA minutes, at age 22 years.

### Outcomes

The primary outcome was change in fat mass index (FMI) from age 18 to 22 years (∆FMI). The secondary outcome was change in fat mass from age 18 to 22 years (∆FM). We also explored change in body mass index (BMI) from age 18 to 22 years (∆BMI) as an outcome. During clinic visits at age 18 and 22 years, participants underwent a whole-body DXA scan (GE Lunar Prodigy, USA). Detailed study procedures have been described elsewhere [29]. Scan images were analyzed using the in-built GE Lunar enCore software. Fat mass (kg) was derived from the image analysis. FMI was calculated by dividing fat mass (kg) by height squared (height^2^).

### Confounders

Several potential confounders were considered: the exact age at the age 22 assessment, wealth index quintiles at age 18 years, education level at age 22 years (0–8, 9–11, or ≥ 12 years of schooling), energy intake quintiles at ages 18 and 22, and FMI at age 18. Wealth index, education level, and energy intake were grouped to allow for non-linear trends in association between the confounders and the outcome. Wealth index was calculated using a principal component analysis based on a set of goods and assets, from which the first component was extracted and then divided into quintiles [30]. Missing data for wealth index quintile (*n* = 2) was imputed to the middle category. Daily energy intake (kcal/day) at ages 18 and 22 years was estimated using a food frequency questionnaire [31]. Daily energy intake (kcal/day) was categorized into five groups based on sex-specific quintile cut-points. Missing data for the energy intake quintile at age 22 years (*n* = 1) was imputed with the energy intake quintile at age 18 years. Energy intake quintile at age 18 years and change in energy intake quintile from age 18 to 22 years were used to account for the energy intake effects.

### Statistical analysis

All analyses were conducted separately by sex using SAS 9.4 (Cary, NC). To address potential selection bias, we conducted Chi-square analyses to compare the frequencies of the key characteristics (i.e., sex, birthweight, maternal education level, and family income level) between those who were included in the analysis and those who were excluded. Descriptive analyses, including means and standard deviations of the exposure and outcome variables, were conducted. Analysis of variance (ANOVA) was conducted to compare the exposure and outcome variables among the six MVPA groups. A multivariable linear regression model was used to estimate ∆FMI by the MVPA group variable (reference group = Y&Y60), adjusting for age, family wealth index quintile at age 18, education level at age 22 years, energy intake quintile at age 18 years, change in energy intake quintile from age 18 to 22 years, and FMI at age 18 years. The analysis was repeated for the ∆FM outcome. The statistical significance level was set at 0.05.

## Results

At age 18 years, 3,308 participants completed the accelerometry assessment and 3,851 completed the DXA assessment. At age 22 years, 2,803 participants completed the accelerometry assessment and 3,318 completed the DXA assessment. A total of 2,099 participants (1,090 females; 40.0% of the original cohort) who had both accelerometer and DXA data at ages 18 and 22 years were included in this data analysis. As presented in Supplementary Table 1, the included sample was similar to the excluded sample in the distributions of sex, birthweight, maternal education level, and family income level. In the included sample, 30.0% had ≥ 12 years of schooling, 36.9% were enrolled in school, 67.3% were employed, and 54.8% lived with parent(s) at age 22 years.

Among males, there were trends that as wealth index and education level were higher, MVPA was lower and FMI was higher (Supplementary Table 2). Among females, there were trends that as wealth index and education level were higher, MVPA were lower (Supplementary Table 3). However, females in the highest wealth index quintile and ≥ 12 years of schooling tended to have lower FMI. Males and females who remained in school from age 18 to 22 years showed a significantly smaller FMI increase than their counterparts who were no longer in school at age 22 years. However, we found no significant difference in ∆FMI between those who changed from living with a parent at age 18 to living without a parent at age 22 years and those living with a parent at both 18 and 22 years of age. Change in height between 18 years (mean = 173.5 cm for males and 160.8 cm for females) and 22 years of age (mean = 174.1 cm for males and 161.0 cm for females) was < 1 cm and was not significantly different across the six MVPA groups. Mean VPA minutes were low at age 22 years: 2 min for both males and females.

Among males, during the 4-year period from age 18 and 22 years, fat mass increased 4.0 kg for Y&Y60, while it increased 6.1 kg for both Y&Y22 and Y&N (Table 1). Among females, fat mass increased 4.6 kg for Y&Y60, 4.3 kg for Y&Y22, and 6.1 kg for Y&N (Table 2).

**Table 1:** Means of MVPA, fat mass, and FMI in the six MVPA groups among males

	Y&Y60 (*n* = 452)	Y&Y22 (*n* = 278)	Y&N (*n* = 37)	N&Y60 (*n* = 69)	N&Y22 (*n* = 127)	N&N (*n* = 46)	*P*-value
	M ± SD	M ± SD	M ± SD	M ± SD	M ± SD	M ± SD	
MVPA 18, min/d	143 ± 68	111 ± 42	94 ± 33	44 ± 13	38 ± 15	34 ± 16	< 0.01
MVPA 22, min/d	117 ± 58	43 ± 10	17 ± 3	107 ± 42	39 ± 11	14 ± 5	< 0.01
MVPA change, min/d	-26 ± 71	-68 ± 44	-77 ± 33	63 ± 43	1 ± 17	-20 ± 15	< 0.01
Fat mass 18, kg	11.0 ± 8.1	12.7 ± 8.6	12.1 ± 8.8	14.3 ± 9.5	15.0 ± 10.1	14.7 ± 9.4	< 0.01
Fat mass 22, kg	15.0 ± 9.6	18.7 ± 10.9	18.3 ± 10.7	16.4 ± 9.6	18.0 ± 11.0	20.3 ± 11.7	< 0.01
Fat mass change, kg	4.0 ± 6.1	6.1 ± 6.3	6.1 ± 5.6	2.1 ± 5.8	3.0 ± 7.4	5.6 ± 8.4	< 0.01
FMI 18, kg/m^2^	3.7 ± 2.6	4.2 ± 2.9	4.0 ± 3.1	4.7 ± 3.1	4.8 ± 3.2	4.7 ± 2.9	< 0.01
FMI 22, kg/m^2^	5.0 ± 3.1	6.2 ± 3.6	6.0 ± 3.7	5.3 ± 3.0	5.8 ± 3.5	6.5 ± 3.7	< 0.01
FMI change, kg/m^2^	1.3 ± 2.0	2.0 ± 2.1	2.0 ± 1.8	0.6 ± 1.8	1.0 ± 2.4	1.8 ± 2.7	< 0.01
BMI 18, kg/m^2^	22.8 ± 3.3	23.3 ± 3.6	22.7 ± 3.5	22.9 ± 4.0	23.4 ± 4.0	23.6 ± 3.8	0.06
BMI 22, kg/m^2^	24.5 ± 3.9	25.3 ± 4.6	24.7 ± 4.7	24.0 ± 3.9	24.7 ± 4.6	25.4 ± 4.5	0.08
BMI change, kg/m^2^	1.7 ± 2.2	2.1 ± 2.3	2.0 ± 2.0	1.1 ± 1.9	1.3 ± 2.6	1.8 ± 3.2	< 0.01

*BMI* Body mass index, *FMI* Fat mass index, *MVPA* Moderate- and vigorous-intensity physical activity, *M* ± *SD* Mean ± standard deviation, *N&N* < 60 min/day at age 18 and < 22 min/day at age 22, *N&Y22* < 60 min/day at age 18 and ≥ 22 min/day at age 22, *N&Y60* < 60 min/day at age 18 and ≥ 60 min/day at age 22, *Y&N* ≥ 60 min/day at age 18 and < 22 min/day at age 22, *Y&Y22* ≥ 60 min/day at age 18 and ≥ 22 min/day at age 22, *Y&Y60* ≥ 60 min/day at age 18 years and ≥ 60 min/day at age 22

**Table 2:** Means of MVPA, fat mass, and FMI in six MVPA groups among females

	Y&Y60 (*n* = 245)	Y&Y22 (*n* = 268)	Y&N (*n* = 50)	N&Y60 (*n* = 79)	N&Y22 (*n* = 314)	N&N (*n* = 134)	*P*-value
	M ± SD	M ± SD	M ± SD	M ± SD	M ± SD	M ± SD	
MVPA 18, min/d	109 ± 45	89 ± 26	87 ± 29	42 ± 13	40 ± 12	34 ± 14	< 0.01
MVPA 22, min/d	92 ± 30	42 ± 11	15 ± 5	80 ± 19	37 ± 10	14 ± 5	< 0.01
MVPA change, min/d	-18 ± 47	-47 ± 28	-72 ± 29	38 ± 22	-3 ± 15	-20 ± 14	< 0.01
Fat mass 18, kg	19.9 ± 8.4	21.6 ± 9.6	20.7 ± 8.5	20.9 ± 7.9	21.5 ± 9.0	22.0 ± 8.8	< 0.01
Fat mass 22, kg	24.5 ± 10.3	25.9 ± 11.4	26.8 ± 11.9	23.7 ± 8.2	24.8 ± 10.2	26.3 ± 10.7	< 0.01
Fat mass change, kg	4.6 ± 5.6	4.3 ± 6.6	6.1 ± 6.1	2.7 ± 5.4	3.3 ± 5.3	4.3 ± 5.4	< 0.01
FMI 18, kg/m^2^	7.7 ± 3.2	8.4 ± 3.8	8.2 ± 3.3	8.1 ± 2.9	8.2 ± 3.4	8.4 ± 3.4	< 0.01
FMI 22, kg/m^2^	9.5 ± 3.9	10.1 ± 4.5	10.6 ± 4.7	9.1 ± 3.0	9.5 ± 3.8	10.1 ± 4.2	< 0.01
FMI change, kg/m^2^	1.8 ± 2.1	1.7 ± 2.6	2.4 ± 2.5	1.0 ± 2.1	1.3 ± 2.0	1.7 ± 2.2	< 0.01
BMI 18, kg/m^2^	23.0 ± 4.1	23.5 ± 4.8	23.3 ± 4.2	22.9 ± 3.8	22.9 ± 4.2	23.0 ± 4.2	0.85
BMI 22, kg/m^2^	25.2 ± 5.0	25.6 ± 5.9	26.0 ± 6.4	24.3 ± 3.8	24.5 ± 4.9	24.9 ± 5.3	0.21
BMI change, kg/m^2^	2.2 ± 2.5	2.0 ± 3.1	2.8 ± 3.4	1.4 ± 2.2	1.6 ± 2.5	1.9 ± 2.6	0.02

*BMI* Body mass index, *FMI* Fat mass index, *MVPA* Moderate- and vigorous-intensity physical activity, *M* ± *SD* Mean ± standard deviation, *N&N* < 60 min/day at age 18 and < 22 min/day at age 22, *N&Y22* < 60 min/day at age 18 and ≥ 22 min/day at age 22, *N&Y60* < 60 min/day at age 18 and ≥ 60 min/day at age 22, *Y&N* ≥ 60 min/day at age 18 and < 22 min/day at age 22, *Y&Y22* ≥ 60 min/day at age 18 and ≥ 22 min/day at age 22, *Y&Y60* ≥ 60 min/day at age 18 years and ≥ 60 min/day at age 22

**Fig. 1:**
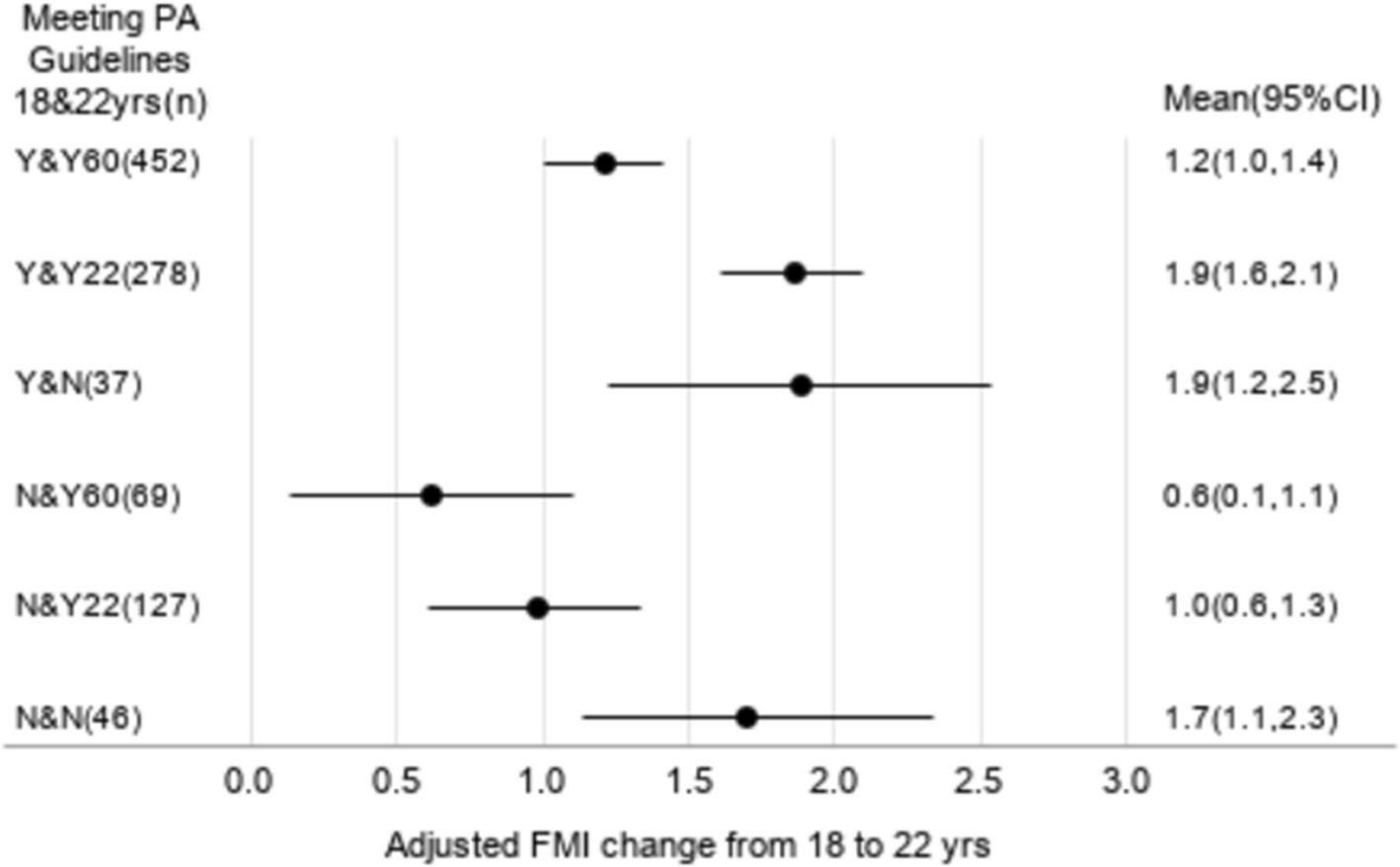
Adjusted means of FMI change (kg/m.^2^) from age 18 to 22 years in six MVPA groups among males. The means of change in FMI were adjusted for age in years, wealth index quintile at age 18 years, years of schooling at age 22 years, energy intake quintile at age 18 years, change in energy intake quintile from age 18 to 22 years, and FMI at age 18 years. FMI, fat mass index; MVPA, moderate- and vigorous-intensity physical activity; PA, physical activity

**Fig. 2:**
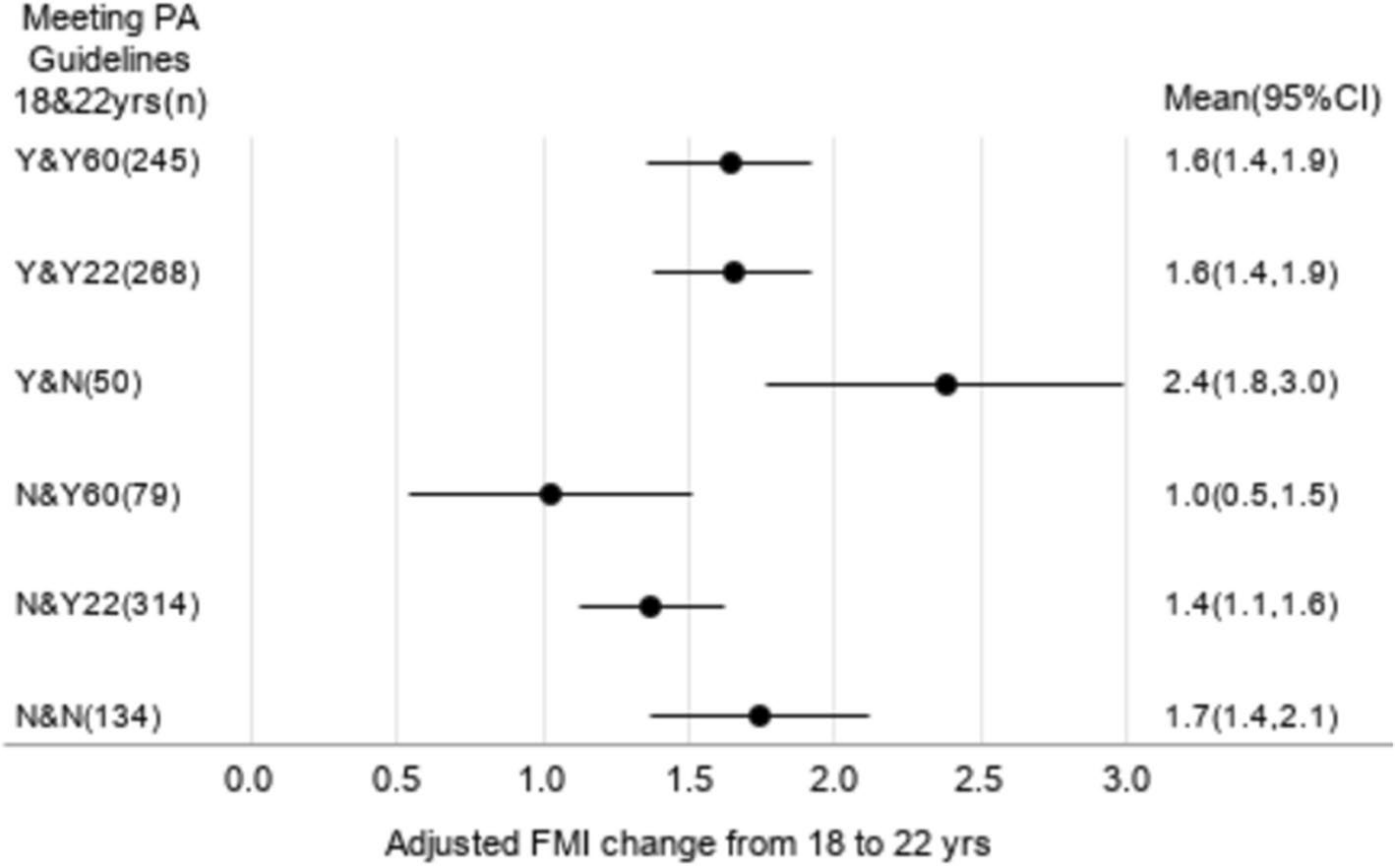
Adjusted means of FMI change (kg/m.^2^) from age 18 to 22 years in six MVPA groups among females. The means of change in FMI were adjusted for age in years, wealth index quintile at age 18 years, years of schooling at age 22 years, energy intake quintile at age 18 years, change in energy intake quintile from age 18 to 22 years, and FMI at age 18 years. FMI, fat mass index; MVPA, moderate- and vigorous-intensity physical activity; PA, physical activity

After adjusting for the covariates among males (Fig. 1), Y&Y22 had a significantly larger increase in FMI compared to Y&Y60. However, ∆FMI did not differ between Y&Y22 and Y&N. Among males who did not meet the youth PAG at age 18 years, FMI increase was the smallest in N&Y60, followed by N&Y22 and N&N. After adjusting for the covariates among females (Fig. 2), ∆FMI did not differ between Y&Y22 and Y&Y60. However, Y&Y22 had a significantly smaller increase in FMI compared to Y&N. Among females who did not meet the youth PAG at age 18 years, FMI increase was the smallest in N&Y60, followed by N&Y22 and N&N.

When ∆FM was examined as an outcome, we found consistent results: males in the Y&Y22 and Y&N group gained 2.0 kg more fat mass compared to males in the Y&Y60 group (Supplementary Figs. 1). Females in the Y&N group gained 1.6 kg more fat mass compared to females in the Y&Y60 or Y&Y22 group (Supplementary Fig. 2). The ∆BMI outcome also showed consistent results: males in the Y&Y22 group had higher ∆BMI than males in the Y&Y60 group and females in the Y&Y60 and Y&Y22 groups had similar ∆BMI (Supplementary Figs. 3 and 4). When wMVPA groups were used as the exposure variable, we found consistent results: larger ∆FMI in Y&Y22 and Y&N than Y&Y60 among males and larger ∆FMI in Y&N than Y&Y22 or Y&Y60 among females (Supplementary Figs. 5 and 6).

## Discussion

We found that among males who were active ≥ 60 min/day of MVPA in late adolescence (age 18 years), those maintaining ≥ 60 min/day of MVPA in early adulthood (age 22 years) had less fat gain compared with those engaged in MVPA 22–59 min/day (meeting the adult PAG). Males who reduced MVPA to 22–59 min/day and those who reduced MVPA 0–21 min/day (not meeting the adult PAG) in early adulthood showed the same amount of fat gain. Among females who were active in late adolescence, those who engaged in ≥ 22 min/day of MVPA in early adulthood (meeting the adult PAG) experiences less fat gain compared with females who reduced MVPA to 0–21 min/day in early adulthood. In addition, for males and females who were inactive in late adolescence, higher MVPA in adulthood was associated with lower fat gain. After accounting for the intensity of PA at age 22 years (wMVPA groups), the findings remained the same.

The substantial difference in recommended MVPA levels between youth and adults might reflect different goals between the groups, i.e., achieving optimal health benefits for youth versus preventing non-communicable diseases for adults [9, 10]. Although these goals may overall be appropriate, the abrupt PAG change for populations in emerging adulthood is poorly justified with little evidence of a dose–response relationship between MVPA and health outcomes during the emerging adulthood period. The present study is one of only a few prospective studies to examine the relationship between MVPA and adiposity during emerging adulthood in a large sample. This study suggests that the currently recommended level of MVPA for adults may be insufficient for lowering fat gain in emerging adulthood among males, while it may be sufficient among females. These findings call for a re-assessment of the current MVPA recommendations for populations in emerging adulthood. As an individual at the end of adolescence is not the same as an adult in terms of physical, physiological, intellectual, social, emotional, and behavioral measures [32], experts in adolescent medicine as well as the United Nations have (re)defined the “youth” period to include up to 24 years of age [32, 33], which adds the emerging adulthood period. Applying the current youth MVPA recommendations to the population in emerging adulthood could be considered. Ultimately, prospective dose–response relationship studies across the emerging adulthood period will help identify how much MVPA is optimal for specific health outcomes to inform future PAG.

In emerging adulthood, most individuals stop growing taller while body fat continues to accumulate. Katsoulis et al. [16] showed that in comparisons of weight gain across U.K. adults from age 18 to 75 years, young adults (18–24 years of age) were identified as having the highest risk of weight gain. The present study is one of the few to examine adiposity development in emerging adulthood in a middle-income country. In the 1993 Pelotas study sample, we found that fat mass continued to increase on average 1.0 kg per year in both males and females throughout the 4 years between age 18 and 22 years. Emerging adulthood is a unique life stage when many significant life events occur, such as leaving the parental home, starting postsecondary education, and/or entering the labor market. Although evidence is lacking to explain why such a large increase in adiposity occurs during emerging adulthood [34], studies in high-income countries found that such life events were associated with obesogenic behaviors. For example, leaving the parental home and moving to college were associated with less healthy diet and PA behaviors [35, 36]. These life events could serve as key opportunities for interventions in young adults, such as improving PA and dietary environment in college [16]. However, the impact of life events that occur during emerging adulthood could be somewhat different in LMICs compared to high-income countries. In the present study sample, those who remained in school from age 18 to 22 years showed *lower* fat gain than their counterparts who were no longer in school at age 22 years, while there was no difference in fat gain between those who changed from living with a parent at age 18 to living without a parent at age 22 years and those living with a parent at both 18 and 22 years of age, as well as between those employed and unemployed. Future research is warranted to better understand fat gain during emerging adulthood, particularly in LMICs.

Although empirical data are limited from LMICs, it appears that the development of body fat is differently influenced by societal factors in high-income countries vs. middle income countries [20]. For example, a prior study reported a negative association between socioeconomic status and fat mass in high-income countries, but a positive or no association in middle-income countries [20]. The present study demonstrated that higher socioeconomic status was associated with higher body fat among young Brazilian men. This positive association could partly be explained by lower MVPA among young Brazilian men in a higher socioeconomic position. However, the relationships were somewhat inconsistent among young Brazilian women: females the highest wealth index quintile had the smallest fat gain between age 18 and 22 years and the lowest absolute fat mass at age 22 years, while they were also the least physically active. Nonetheless, after accounting for socioeconomic status in our analysis, we found that achieving ≥ 60 min/day of MVPA at ages 18 and 22 years was associated with a lower gain in fat mass compared to those who reduced MVPA to < 22 min/day at age 22 years. The benefits of MVPA on body fat gain should be widely communicated and advocated in the general population.

Some limitations should be acknowledged. First, despite an overall large sample size, the Y&N group accounted for only a small fraction of the sample (≤ 50 participants; ≤ 2.4%), producing wide CIs. Second, we cannot rule out that higher fat mass could have influenced lower MVPA (“reverse causation”) [37]. Third, use of two different types of accelerometers and different sampling frequencies could have provided different MVPA estimates. Further, wrist accelerometer-measured PA metrics may not be comparable with hip accelerometer-measured PA metrics. Fourth, this study did not account for potential seasonal effects. Lastly, the results may not be generalizable to the entire 1993 Pelotas study sample, because only 40% of the original sample was included in the current data analysis, which may have introduced bias in our findings. In addition, the results may not be generalizable young adults from other populations in different settings.

## Conclusions

Our findings suggest that among individuals who were active in late adolescence, engaging in ≥ 22 min/day of MVPA in adulthood (meeting the adult PAG) is associated with lower body fat gain for females, but not for males.

## Availability for data and materials

Data may be available upon request to Ana MB Menezes (anamene.epi@gmail.com) at Federal University of Pelotas, Brazil.

## Supplementary Information

**Additional file 1:** **Supplementary Table 1.** Comparisons of characteristics of the included sample and the excluded sample. **Supplementary Table 2.** Means of MVPA and FMI by wealth index and education level among males. **Supplementary Table 3.** Means of MVPA and FMI by wealth index and education level among females. **Supplementary Figure 1.** Adjusted means of the change in fat mass (kg) from age 18 to 22 years by six MVPA groups among males. **Supplementary Figure 2.** Adjusted means of the change in fat mass (kg) from age 18 to 22 years by six MVPA groups among females. **Supplementary Figure 3.** Adjusted means of the change in BMI from age 18 to 22 years by six MVPA groups among males. **Supplementary Figure 2.** Adjusted means of the change in BMI from age 18 to 22 years by six MVPA groups among females. **Supplementary Figure 5.** Adjusted means of the change in fat mass (kg) from age 18 to 22 years by six wMVPA groups among males. Supplementary Figure 6. Adjusted means of the change in fat mass (kg) from age 18 to 22 years by six wMVPA groups among females.

## Abbreviations

∆BMI: Change in body mass index
∆FM: Change in fat mass
∆FMI: Change in fat mass index
BMI: Body mass index
DXA: Dual-energy X-ray absorptiometry
FM: Fat mass
FMI: Fat mass index
LMIC: Low- to middle-income country
MPA: Moderate-intensity physical activity
MVPA: Moderate- and vigorous-intensity physical activity
N&N: Not meeting the youth MVPA guideline at age 18 years (< 60 min/day) and the adult MVPA guideline at age 22 years (< 22 min/day)
N&Y22: Not meeting the youth MVPA guideline at age 18 years (< 60 min/day) and meeting the adult MVPA guideline at age 22 years (≥ 22 min/day)
N&Y60: Not meeting the youth MVPA guideline at age 18 years (< 60 min/day) and meeting the youth MVPA guideline at age 22 years (≥ 60 min/day)
Y&N: Meeting the youth MVPA guideline at age 18 years (≥ 60 min/day) and not meeting the adult MVPA guideline at age 22 years (< 22 min/day)
Y&Y22: Meeting the youth MVPA guideline at age 18 years (≥ 60 min/day) and the adult MVPA guideline at age 22 years (≥ 22 min/day)
Y&Y60: Meeting the youth MVPA guideline at age 18 years (≥ 60 min/day) and meeting the youth MVPA guideline at age 22 years (≥ 60 min/day)
PA: Physical activity
PAG: Physical activity guidelines
VPA: Vigorous-intensity physical activity
WHO: World Health Organization
wMVPA: Weighted moderate- and vigorous-intensity physical activity
