# Supplementary material for: Longitudinal change in physical activity and adiposity in the transition from adolescence to early adulthood: the 1993 Pelotas cohort study
Source: Int J Behav Nutr Phys Act. 2022 Jul 14;19:83. doi: 10.1186/s12966-022-01321-0 (PMC9284823; doi:10.1186/s12966-022-01321-0)
Supplement: Supplementary file 1 — Additional file 1: Supplementary Table 1. Comparisons of characteristics of the included sample and the excluded sample. Supplementary Table 2. Means of MVPA and FMI by wealth index and education level among males. Supplementary Table 3. Means of MVPA and FMI by wealth index and education level among females. Supplementary Figure 1. Adjusted means of the change in fat mass (kg) from age 18 to 22 years by six MVPA groups among males. Supplementary Figure 2. Adjusted means of the change in fat mass (kg) from age 18 to 22 years by six MVPA groups among females. Supplementary Figure 3. Adjusted means of the change in BMI from age 18 to 22 years by six MVPA groups among males. Supplementary Figure 2. Adjusted means of the change in BMI from age 18 to 22 years by six MVPA groups among females. Supplementary Figure 5. Adjusted means of the change in fat mass (kg) from age 18 to 22 years by six wMVPA groups among males. Supplementary Figure 6. Adjusted means of the change in fat mass (kg) from age 18 to 22 years by six wMVPA groups among females. [file 12966_2022_1321_MOESM1_ESM.docx]

**Supplementary Data**

Supplementary Table 1. Comparisons of characteristics of the included sample and the excluded sample

|  | Included sample | Excluded sample | P-value |
| --- | --- | --- | --- |
|  | n (%) | n (%) |  |
| Total | 2,099 (40.0) | 3150 (60.0) |  |
| Sex |  |  | 0.07 |
| Males | 1009 (38.8) | 1594 (61.2) |  |
| Females | 1090 (41.2) | 1555 (58.8) |  |
| Birthweight |  |  | 1.00 |
| <2500 | 204 (40.0) | 306 (60.0) |  |
| ≥2500 | 1895 (40.0) | 2844 (60.0) |  |
| Maternal education |  |  | 0.69 |
| ≤7 years | 1553 (39.8) | 2346 (60.2) |  |
| ≥8 years | 546 (40.4) | 804 (59.6) |  |
| Family income (minimal wages) |  |  | 0.64 |
| <4 | 1421 (40.2) | 2113 (59.8) |  |
| ≥4 | 678 (39.5) | 1037 (60.5) |  |

Supplementary Table 2. Means of MVPA and FMI by wealth index and education level among males

|  | Sample size, n | MVPA at age 18, min/d | MVPA at age 22, min/d | FMI at age 18, kg/m^2^ | FMI at age 22, kg/m^2^ |
| --- | --- | --- | --- | --- | --- |
| Wealth index quintile at age 18 |  |  |  |  |  |
| 1 (lowest) | 165 | 129±72 | 94±70 | 3.4±2.5 | 4.4±3.0 |
| 2 | 217 | 128±77 | 92±65 | 3.6±3.0 | 5.1±3.6 |
| 3 | 192 | 107±53 | 80±54 | 4.1±2.8 | 5.7±3.3 |
| 4 | 242 | 97±61 | 70±45 | 4.4±3.0 | 6.0±3.5 |
| 5 (highest) | 193 | 80±51 | 55±43 | 4.8±2.8 | 6.2±3.2 |
| Education level at age 22 |  |  |  |  |  |
| 0-8 years | 362 | 131±75 | 98±68 | 3.5±2.9 | 4.9±3.6 |
| 9-11 years | 413 | 105±59 | 74±52 | 4.3±2.9 | 6.0±3.4 |
| ≥12 years | 234 | 75±45 | 52±31 | 4.6±2.7 | 5.8±3.0 |

FMI, fat mass index; MVPA, moderate- and vigorous-intensity physical activity.

Supplementary Table 3. Means of MVPA and FMI by wealth index and education level among females

|  | Sample size, n | MVPA at age 18, min/d | MVPA at age 22, min/d | FMI at age 18, kg/m^2^ | FMI at age 22, kg/m^2^ |
| --- | --- | --- | --- | --- | --- |
| Wealth index quintile at age 18 |  |  |  |  |  |
| 1 (lowest) | 251 | 79±46 | 56±34 | 8.3±3.6 | 10.2±4.3 |
| 2 | 198 | 74±36 | 51±31 | 8.1±3.3 | 9.8±3.8 |
| 3 | 216 | 68±43 | 51±35 | 8.3±3.6 | 10.0±4.3 |
| 4 | 228 | 67±41 | 48±32 | 8.0±3.2 | 9.6±4.0 |
| 5 (highest) | 197 | 54±30 | 41±26 | 8.0±3.3 | 8.9±3.7 |
| Education level at age 22 |  |  |  |  |  |
| 0-8 years | 232 | 78±50 | 55±36 | 8.4±3.9 | 10.3±4.5 |
| 9-11 years | 462 | 73±40 | 51±35 | 8.2±3.4 | 10.0±4.1 |
| ≥12 years | 396 | 60±33 | 45±26 | 8.0±3.1 | 9.1±3.7 |

FMI, fat mass index; MVPA, moderate- and vigorous-intensity physical activity.

Supplementary Figure 1. Adjusted means of the change in fat mass (kg) from age 18 to 22 years by six MVPA groups among males


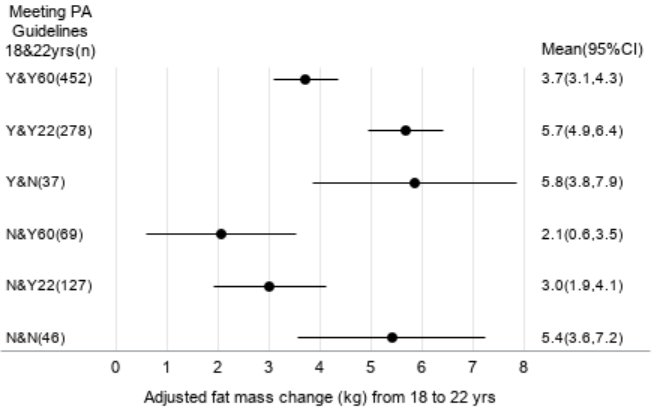


The means of change in fat mass were adjusted for age in years, wealth index quintile at age 18 years, years of schooling at age 22 years, energy intake quintile at age 18 years, change in energy intake quintile from age 18 to 22 years, and FMI at age 18 years.

FMI, fat mass index; MVPA, moderate- and vigorous-intensity physical activity; PA, physical activity.

Supplementary Figure 2. Adjusted means of the change in fat mass (kg) from age 18 to 22 years by six MVPA groups among females


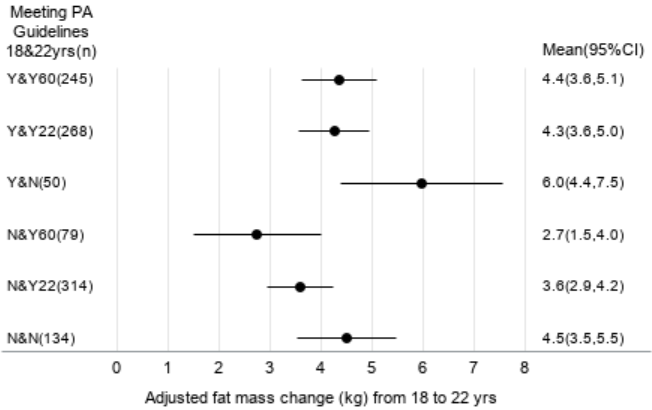


The means of change in fat mass were adjusted for age in years, wealth index quintile at age 18 years, years of schooling at age 22 years, energy intake quintile at age 18 years, change in energy intake quintile from age 18 to 22 years, and FMI at age 18 years.

FMI, fat mass index; MVPA, moderate- and vigorous-intensity physical activity; PA, physical activity.

Supplementary Figure 3. Adjusted means of the change in BMI from age 18 to 22 years by six MVPA groups among males


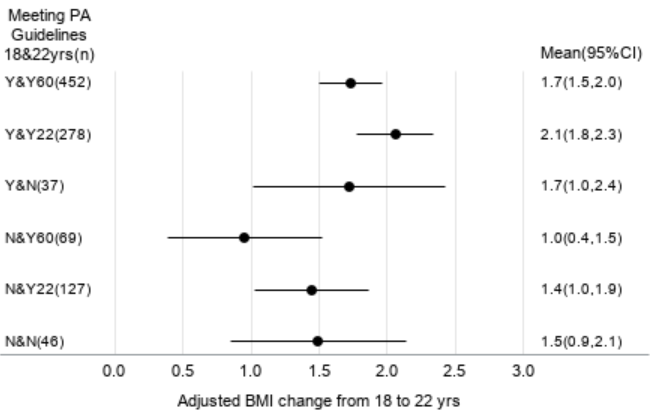


The means of change in fat mass were adjusted for age in years, wealth index quintile at age 18 years, years of schooling at age 22 years, energy intake quintile at age 18 years, change in energy intake quintile from age 18 to 22 years, and BMI at age 18 years.

BMI, body mass index; MVPA, moderate- and vigorous-intensity physical activity; PA, physical activity.

Supplementary Figure 2. Adjusted means of the change in BMI from age 18 to 22 years by six MVPA groups among females


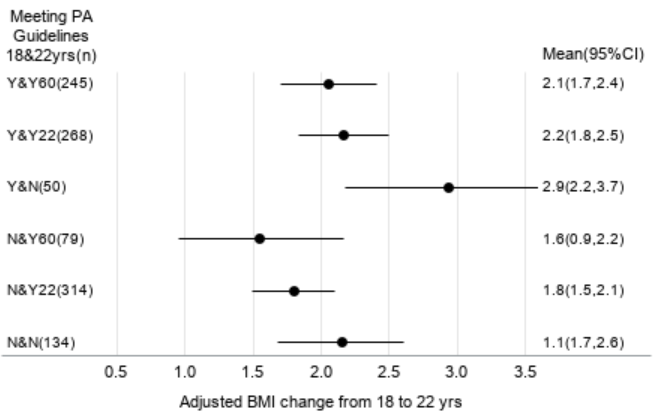


The means of change in fat mass were adjusted for age in years, wealth index quintile at age 18 years, years of schooling at age 22 years, energy intake quintile at age 18 years, change in energy intake quintile from age 18 to 22 years, and BMI at age 18 years.

BMI, body mass index; MVPA, moderate- and vigorous-intensity physical activity; PA, physical activity.

Supplementary Figure 5. Adjusted means of the change in fat mass (kg) from age 18 to 22 years by six wMVPA groups among males


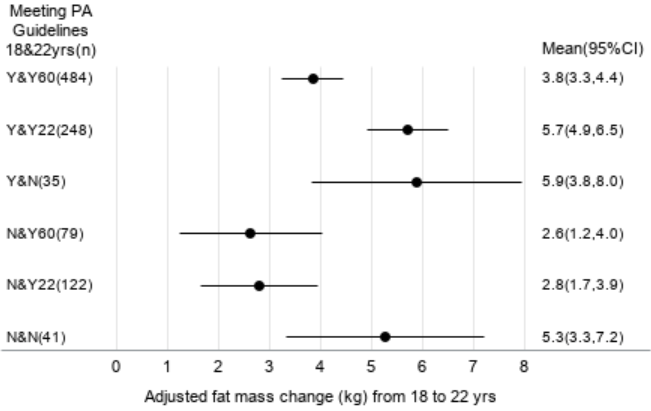


To define wMVPA groups, MPA-equivalent minutes at age 22 years were used.

The means of change in fat mass were adjusted for age in years, wealth index quintile at age 18 years, years of schooling at age 22 years, energy intake quintile at age 18 years, change in energy intake quintile from age 18 to 22 years, and FMI at age 18 years.

FMI, fat mass index; MPA, moderate-intensity physical activity; wMVPA, weighted moderate- and vigorous-intensity physical activity; PA, physical activity.

Supplementary Figure 6. Adjusted means of the change in fat mass (kg) from age 18 to 22 years by six wMVPA groups among females


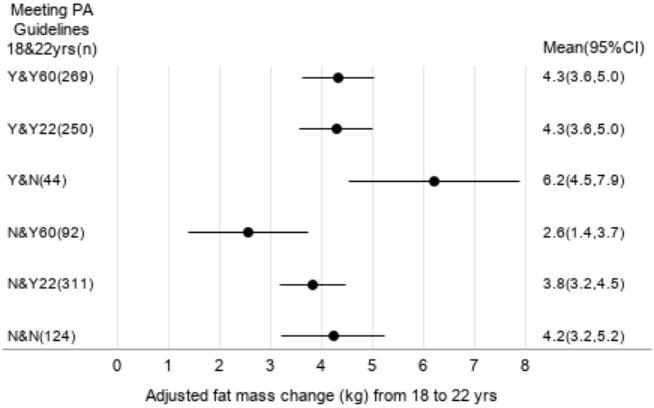


To define wMVPA groups, MPA-equivalent minutes at age 22 years were used.

The means of change in fat mass were adjusted for age in years, wealth index quintile at age 18 years, years of schooling at age 22 years, energy intake quintile at age 18 years, change in energy intake quintile from age 18 to 22 years, and FMI at age 18 years.

FMI, fat mass index; MPA, moderate-intensity physical activity; wMVPA, weighted moderate- and vigorous-intensity physical activity; PA, physical activity.
